# MicroRNA in extracellular vesicles regulates inflammation through macrophages under hypoxia

**DOI:** 10.1038/s41420-021-00670-2

**Published:** 2021-10-11

**Authors:** Ye Li, Jin Tan, Yuyang Miao, Qiang Zhang

**Affiliations:** 1 Department of Geriatrics, Tianjin Geriatrics Institute, Tianjin Medical University General Hospital, 300052 Tianjin, China; 2 Tianjin Medical University, 300052 Tianjin, China

**Keywords:** Inflammation, Cell polarity, Molecular biology

## Abstract

Extracellular vesicle (EV), critical mediators of cell-cell communication, allow cells to exchange proteins, lipids, and genetic material and therefore profoundly affect the general homeostasis. A hypoxic environment can affect the biogenesis and secrete of EVs, and the cargoes carried can participate in a variety of physiological and pathological processes. In hypoxia-induced inflammation, microRNA(miRNA) in EV participates in transcriptional regulation through various pathways to promote or reduce the inflammatory response. Meanwhile, as an important factor of immune response, the polarization of macrophages is closely linked to miRNAs, which will eventually affect the inflammatory state. In this review, we outline the possible molecular mechanism of EV changes under hypoxia, focusing on the signaling pathways of several microRNAs involved in inflammation regulation and describing the process and mechanism of EV-miRNAs regulating macrophage polarization in hypoxic diseases.

## Abstract (web summary)

EV-miRNAs regulate the polarization phenotype of macrophages and promote the release of inflammation-related cytokines through a variety of signaling pathways under hypoxia, ultimately affect the development of inflammation.

## Graphical abstract

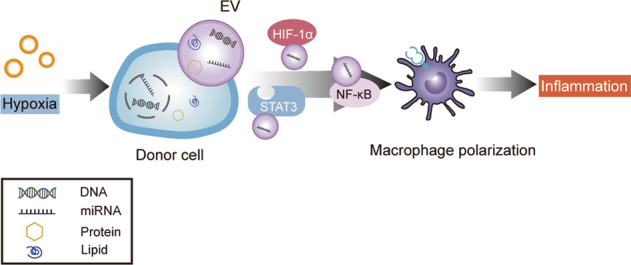

## Facts

- Hypoxia contribute to changes in the biogenesis of EVs, such as increased number of secretion and alterations in cargo sorting, and multiple molecular mechanisms are involved, including HIFs, the Rab family of proteins and oxidative stress.
- As one of the cargoes of EVs, miRNAs can promote or inhibit the polarization of macrophages to M1 or M2 phenotypes, respectively, thus influencing the inflammatory state.
- EV-miRNAs are closely related to hypoxia and inflammation, and the most prominent are miR-233, miR-23a, and miR-21, which can be used as a potential regulator to adjust the function of receptor cells.
- NF-κB, TLR, and STAT3 signaling are intertwined into a network that links miRNAs function to inflammatory events.

## Open questions

- What is the key mechanism that affects the increased secretion of EVs, selective cargo sorting and loading under hypoxia?
- Are there other more critical EV-miRNAs involved in the progression of inflammation under hypoxia by regulating the polarization of macrophages?
- What is the key signaling pathway that EV-miRNA regulates macrophages to control inflammation under hypoxia?
- Whether it’s possible to use the characteristics of EVs to manipulate the expression of miRNA for the precise treatment of hypoxic inflammation-related diseases?

## Introduction

Hypoxia is a condition in which the tissue oxygen level can’t satisfy the metabolic demand of cells, resulting in abnormal metabolism and function of cells, and it can exist in a variety of physiological and pathological conditions. To adapt to the hypoxic environment, the cell initiates the hypoxia response mechanism. However, it will cause fatal damage to the cell if cell fails to adapt to more severe hypoxia.

Inflammation is often induced when cells are exposed to hypoxia, and a series of metabolic changes occur in immune cells, mainly including the increase of glycolysis rate and oxygen consumption, which promote the formation of inflammatory mediators [1]. Due to the increased oxygen consumption reduces the utilization rate of local oxygen, inflammation manufactures hypoxia in local tissue more severely. Therefore, hypoxia-induced inflammation creates a metabolic microenvironment that regulates immune and inflammatory transcriptional regulators [2]. Macrophages are formed through the differentiation of monocytes and are the key factors of the immune response, especially to the resolution of inflammation [3].

As a new medium of communication between cells, EVs can transfer cargoes between cells, and contribute to the crosstalk between hypoxia and inflammation. MiRNAs, as one of cargoes of EV, can be used as a post-transcriptional regulator to control many pathophysiological events. Interestingly, miRNAs have been shown to regulate macrophage polarization and downstream inflammatory effect [4–6]. In this review, we discuss the possible mechanisms that EV-miRNAs affect inflammation through macrophage under hypoxia.

## Extracellular vesicles and macrophages under hypoxia

### Physiological and pathological hypoxia

Most tissues can be supplied with oxygen levels that exceed the basal metabolic demands through capillaries, which also satisfy the bioenergy requirements of mitochondria. However, the physiological oxygen concentration in special tissues is relatively low compared with other tissues, which is called “physiological hypoxic” area, such as bone marrow, lymphoid, intestinal mucosa, and placenta [2]. These tissues are characterized by enhancement of cell proliferation and metabolism, whereas with stable and consistent oxygen gradient, which can initiate the physiological hypoxia response but will not destroy the normal tissue structure. This hypoxia response regulates innate and adaptive immunity largely via the oxygen-sensitive transcriptional regulator hypoxia-inducible factor (HIF). HIF promotes physiological responses to hypoxia through the synthesis and secretion of factors (e.g., hepatocellular HIF2-dependent production of erythropoietin [7]; HIF1 induces expression of epithelial-specific barrier protective genes in intestinal epithelial cells [8]). Sustained and moderate hypoxia in these areas is important for effective immune activity in healthy individuals, and physiological hypoxia regulates immune homeostasis through the control of resident immune cells. Hypoxia and microenvironmental cues combined to regulate the level of HIF activity and thereby regulate metabolism and effector sequelae in downstream immune cells. These effects ultimately determine the physiological/pathological response.

In pathological hypoxia (e.g., tumors, obesity, and infected tissues), tissues cannot get a normal level of oxygen supply, which activate immune cells pathological hypoxia responses. Since the metabolic needs of tissues can’t be met through the blood supply, which easily leads to the destruction of physiological functions. Therefore, tissue is exposed to the chaotic and intermittent oxygen gradient. Compared with physiological hypoxia, the consequences of this exposure are more serious, such as inflammation, oxidative stress, and even cell death [9]. A variety of oxygen-sensitive immune-related signaling pathways are activated to affect physiological homeostasis, especially the HIF pathway [2]. These areas contain resident immune cells or recruit them from oxygen-rich bloodstream. HIF affects the downstream function of immunity and inflammation by changing the metabolic state of immune cells. In turn, increased immune activity was associated with signaling levels of HIF activation. Inflammatory products can regulate the state of HIF pathway, such as cytokines, ROS, and nitric oxide. Acute inflammation is characterized by the accumulation of oxygen-consuming neutrophils, which is representative of pathological hypoxia [10]. This “inflammatory hypoxia” is caused by increased oxygen consumption, local cell proliferation, and the migration of various inflammatory cells. Importantly, chronic inflammation of pathologically hypoxic sites may be associated with the development of cancer. Broadly speaking, there is an inextricable link between hypoxia and inflammation.

### The biogenesis of EVs under physiological hypoxia

**Fig. 1:**
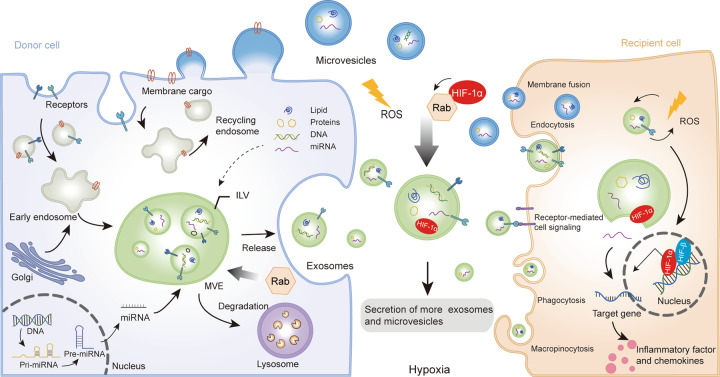
Biogenesis, secretion, and uptake of EVs. Microvesicles are generated after formation by budding from the plasma membrane. Exosomes are essentially intraluminal vesicles(ILV), which are formed in multivesicular endosomes (MVE). Cargoes sorting to MVEs originate from endocytosis at the plasma membrane or to early sorting endosomes. Proteins are transported from the Golgi or internalized from the cell surface, and nucleic acid can be endocytosed and transferred into the early endosomes. Then early endosomes maturate into MVE, which follows either the secretory or degradative pathway. Microvesicles are secreted upon the fusion of MVEs with the cytomembrane. Extracellular vesicles (EVs) may also be internalized by multiple pathways, such as direct signaling through ligand/receptor molecules, the direct fusion of their membrane, endocytosis, macropinocytosis, and even phagocytosis. MicroRNA carried by EVs can uptake into receptor cells and combine with target genes to regulate inflammatory response. Hypoxia can activate HIF-1α signaling and alter the biogenesis of EV, and Rab GTPases and ROS are also involved in this process.

In line with the existing scientific research, EVs can be divided into two types according to size and biogenesis, including microvesicle (MV) and exosome [11] (Fig. 1). The size of MV ranges from 50 nm to 1000 nm, whereas exosome diameter is less than 150 nm [11]. MV is generated by the outward budding, which released vesicles into the extracellular after fission of the plasma membrane. The biogenesis of exosomes mainly consists of the generation of MVEs, cargo sorting, and releasing [12]. Exosomes are essentially intraluminal vesicles(ILV), which are formed by endosomal membrane inward budding during the mature processing of multivesicular endosomes (MVEs), and secreted when MVEs fusion with the cell surface [13]. EV cargo sorting mainly includes two ways: the endosomal sorting complex required for transport (ESCRT)-dependent and the independent mechanisms. The ESCRT family is crucial in cargo sorting and has been reviewed previously [14–16]. Members of the ESCRT family are involved in mediating the loading of proteins or RNA into exosomes [17]. They can add post-translational modifications to control ubiquitination protein sorting to ILV [18]. However, consumption of ESCRT components reduces exosome secretion, indicating that there are ESCRT-independent pathways in cargo sorting. Tetraspanins are fundamental to the composition of ESCRT-independent exosomes, which are more broadly involved in the sorting and loading of proteins, lipids, DNA, and RNA [12]. In addition to carrying waste produced by cells, EVs possess a communication function, packaging parent cell information to target cells, such as proteins, lipids, and genetic materials (including DNA, mRNA, and miRNA) [13].

The placenta is in a mild physiological hypoxic environment during early pregnancy, accompanied by changes in the placental EVs cargo, especially the level of miRNA expression. Studies have confirmed that the influence of miRNAs and protein expression in EVs depends on the types and invasiveness of trophoblast cells [19]. Trophoblast cells express many immunomodulatory proteins that regulate the function of maternal immune cells [20]. It has been found that EVs-enriched-miR-517a-3p secreted by trophoblast cells are delivered to T cells and NK cells to regulate immune effects. In addition, EVs derived from bovine placenta transfer miR-499 to endometrial epithelial cells to inhibit the activation of NF-κB and inflammatory response [21]. This physiological hypoxia-induced immune response and microenvironment is necessary to maintain pregnancy. Although there are other researches on physiological hypoxia, this review mainly focuses on the impact of pathological hypoxia on EVs.

### The effect of hypoxia on the biogenesis of EVs and its molecular mechanism

At present, there’s plenty of research has identified that hypoxia affects EV biogenesis and downstream function, such as the amount of EVs secreted increases in various hypoxic diseases [22, 23]. Interestingly, the nanoparticle tracking analysis shows that hypoxia just stimulates an increase in the number of EV releases, but doesn’t change the size of EVs [22]. Hypoxia can stimulate the rearrangement of the cytoskeleton and extracellular matrix of EV, which leading to reprogramming of recipient cells [24]. Although the mechanism is not fully understood, we mainly discuss several elite roles that may be involved in this process.

#### HIFs participate in the change of EVs cargo sorting and releasing

HIFs are transcription factors that consist of an oxygen-regulated α subunit and a constitutively expressed nuclear β subunit and mediate the adaptation to hypoxia in cells and tissues. HIFs were inhibited when oxygen is sufficient and activated under hypoxia. In hypoxia conditions, accumulating HIF-1α translocates to the nucleus, then associates with HIF-1β and the coactivators p300/CBP, which lead to transcriptional induction by binding to the conserved hypoxia-responsive element [25]. Evidence shows that HIFs were involved in regulating the level of miRNA sorting in EV. It has been found that hypoxia provokes the secretion of miR-100-enriched-EVs by breast cancer cells through HIF-1α, indicating that the releasing of EVs is HIF-dependent [26]. Li et al. identified that renal tubular epithelial cells release more miR-23a-laden EVs and participate in renal tubulointerstitial inflammation through the regulation of HIF-1α in response to hypoxia [27]. Moreover, mitochondrial reactive oxygen species (ROS) induce the increase of HIF-1α activity, thus promoting the release of EVs with pro-inflammatory activity [28]. Meanwhile, EVs also trigger the activity of HIF-1α and mediates the release of cytokines from macrophages. Therefore, HIF-1α and EVs play a synergistic role in regulating the process of immune inflammation. However, the beneficial or harmful effects of hypoxia-driven EV production on recipient cells are very complex and difficult to determine. At present, most studies simply explain that HIF-1α participates in the regulation process of EVs biogenesis under hypoxia, but the exact mechanism needs to be further explored.

#### The Rab GTPases are an important component of intracellular trafficking of EVs

The core of ensuring that cargoes get to the right destination is Rab GTPases, a family of small GTPases of various types that regulate vesicle budding, movement, and fusion by recruiting effector proteins [29]. For example, HIFs mediate the induction of Rab22 and Rab20 to participate in the production and release of EVs [30]. Furthermore, Rab5 and Rab7 are also involved in cargo sorting to early endosomes [31]. As one of the important participants in cytoskeletal and sub-membranous actin rearranging in vesicular secretion, an increase in hypoxic-driven EVs release was observed in B cells when RAB27A binds to HIF1-α [11, 32]. Hypoxia drives activation of signal transducers and activators of transduction-3 (STAT3) pathway, which regulates Rab7 and RAB27A proteins to promote the release of EVs from ovarian cancer cells. However, the mechanism of Rab regulates the plasma fusion or the secretion of MVEs driven by hypoxia, which still needs to be further clarified at present. It has been confirmed that the accumulation of Rab5 around the perinuclear region is higher under hypoxia conditions, which mediates the increase of exosomes secretion of PCa cells [33]. These results demonstrate that the biogenesis of EVs under hypoxia is closely related to the movement of cell membrane and the rearrangement of cytoskeleton.

#### Changes in EV cargo as a link between oxidative stress and inflammation

Oxidative stress is the imbalance between free radicals and antioxidants in the body under hypoxia. The oxidation and redox process will affect the sorting of cargoes, which may also change the composition of EV membrane lipids [23]. As rapidly available substrates for peroxidation, MVs are loaded with oxidizing compounds and released to recipient cells to activate and amplify inflammation [34]. Hedlund et al. reveal that the dysfunction of NK cells in T and B cell leukemia/lymphoma may be due to oxidative stress stimulating the cells to release more immunosuppressive exosomes, resulting in a stronger cytotoxic response [35]. Therefore, oxidative stress in the microenvironment regulates the activity of immune cells through EVs, thus changing the downstream immune function. Interestingly, oxidative stress also can be regulated by exosomes in turn. Mesenchymal stem cell (MSC)-derived exosomes can alleviate the oxidative stress injury of ischemia-reperfusion(I/R) myocardial cells and activate the PI3K/Akt signaling pathway to enhance myocardial function and vitality and reduce systemic inflammation [36]. These direct and indirect shreds of evidence suggest that the hypoxia-driven EVs are closely associated with inflammation and immune response, nevertheless the underlying mechanism needs to be further elucidated.

### Hypoxia and macrophage polarization

**Fig. 2:**
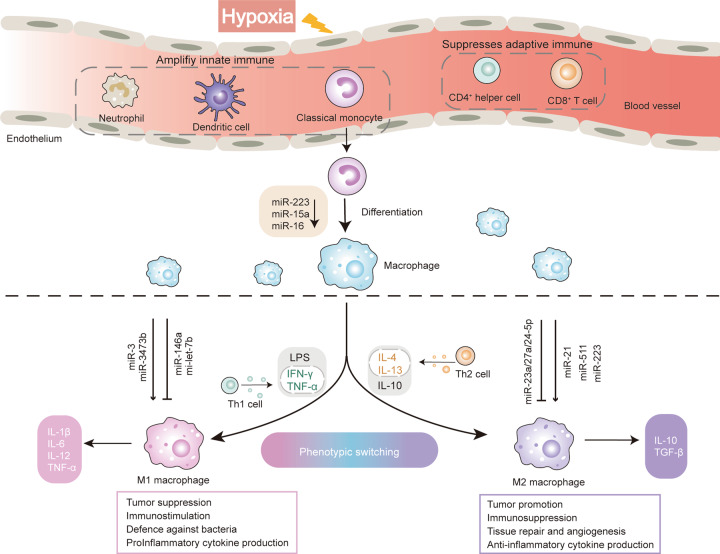
The regulation of miRNA to macrophage polarization. Hypoxia suppresses the adaptive immune system response, and amplifies the activity of innate immune cells, partly by negatively regulating the polarization of CD4 + helper T cells to prevent over-activation of the immune host defense system. Macrophages are formed through the differentiation of monocytes and could be divided schematically into two main classes: M1 and M2. Monocytes mature to M1 macrophages induced by IFN-γ, TNF-α, and LPS, which secrete pro-inflammatory cytokines, such as TNF-α, IL-1β, IL-6, IL-12. They are characterized by the cytotoxic activity against bacteria and tumor cell, which usually strengthens inflammation. M2 macrophages polarization induced by IL-4, IL-13, and IL-10, which usually shut down destructive immune system activation by producing anti-inflammatory cytokines IL-10 and TGF-β. M2 macrophages participate in wane inflammation, scavenge debris and tumor promotion and restore tissue repair. In the process of human monocyte-macrophage differentiation, the expression of miR-223, miR-15a, and miR-16 is significantly decreased, which can promote the polarization of M1 macrophages. MiRNAs expressed at different levels in response to environmental stimuli can participate in the polarization of macrophages to M1 or M2 phenotypes, which can promote or inhibit the polarization, respectively.

Macrophages are formed through the differentiation of monocytes and are the key factors of immune response. Macrophages have been artificially divided into two different activated states [37] (Fig. 2). In recent years, the definition of M1/M2 has been re-evaluated, but their view that macrophages are a set of intermediate phenotypes rather than two opposite extremes has not been widely accepted. M1 macrophages can be induced by multiple inflammatory mediators, mainly including interferon-γ (IFN-γ), tumor necrosis factor (TNF-α), and bacterial lipopolysaccharide (LPS) through TLR, and secrete higher levels of pro-inflammatory cytokines, such as TNF-α, IL-1β, IL-6, IL-12 [38]. M2 macrophages usually participate in wane inflammation and restore tissue repair, polarize by IL-4, IL-13, and IL-10. It can shut down destructive immune system activation by producing anti-inflammatory cytokines such as IL-10 and TGF-β [3]. The bioenergetics nature of macrophages is glycolytic cells, which polarization and function require reprogramming of intracellular metabolism [39]. Polarized macrophages can exhibit unique characteristics of glucose metabolism [40]. For example, M1 macrophages are manifested as increased glucose consumption and lactate release and tend to switch to anaerobic glycolysis pathways to meet their large and rapid energy needs, while M2 macrophages mainly exhibit oxidative glucose metabolism pathways, which produces higher amounts of ATP by oxidative phosphorylation than by glycolysis alone [41, 42]. These metabolic differences may occur due to differences in inflammatory dynamics and duration. M1 macrophages rapidly produce ATP through anaerobic glycolysis in response to a high-intensity infection. On the other hand, in order to deal with persistent inflammation and eventually resolve the inflammation requires the M2 macrophages to produce ATP efficiently but slowly [43].

**Fig. 3:**
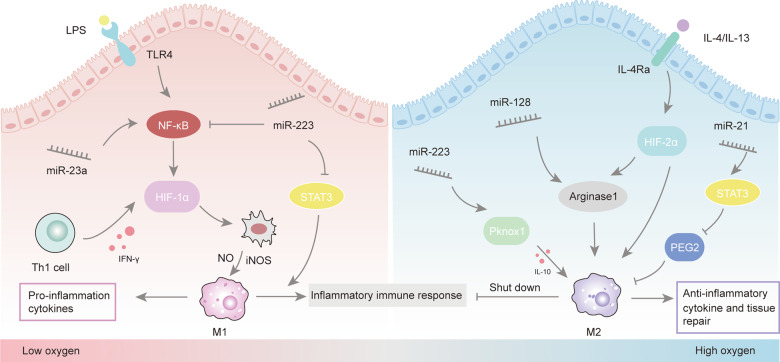
Different HIF subtypes affect the polarization phenotype of macrophages. M1 signals such as IFN-γ and LPS promote the expression of HIF-1α, while M2 signals such as IL-4 and IL-13 facilitate the accumulation of HIF-2α. TLR4 was highly expressed in infiltrating macrophages, while the M2 response stimulated by IL-4 was inhibited under hypoxia. HIF-1α drives the expression of M1 macrophage, while HIF-2α is more closely related to M2 macrophage. Different miRNA can regulate the polarization of macrophages through the HIF pathway and affect the occurrence and development of inflammation.

Pathological hypoxia often leads to chronic persistent inflammation, which can change tissue microenvironment and external factors to affect the process of macrophage polarization. The killing effect of macrophages is HIF-dependent during hypoxia and HIF subtypes meant a great deal to function of macrophages [44, 45]. The levels of HIF-1α and HIF-2α are regulated by Th1/M1 and Th2/M2 cytokines, respectively. M1 signals such as IFN-γ and LPS promote the expression of HIF-1α, while M2 signals such as IL-4 and IL-13 facilitate the accumulation of HIF-2α [46] (Fig. 3). And these different impacts are bound up with the metabolism of macrophages. During the activation of M1 macrophages induced by LPS, the activity of the tricarboxylic acid cycle (TCA) decreased, but the TCA intermediate succinate increased significantly, which can stabilize HIF1-α and induce production of IL-1β [47]. While the expression of HIF-2α in M2 macrophages induces the production of arginase 1 (Arg1) and inhibits the generation of NO [48]. The phagocytosis of macrophages under hypoxia depends on HIF-1α, but HIF-2α is not necessary. The production of inducible nitric oxide synthase (iNOS) and NO are vital factors influencing M1 macrophages bactericidal and tumor-killing activity [49], while the Arg1 expressed by M2 macrophages is mainly involved in cell proliferation, tumor promotion, and tissue remodeling [50]. In addition to glucose metabolism, fatty acids, vitamins, and iron metabolism are also take part in macrophage polarization [40]. Nevertheless, the specific metabolic pathways of macrophages have not yet been fully clarified and need to be resolved in the future.

MiRNA supports a crucial role in the reprogramming process of macrophages, as a switch, which determines the ultimate pro/anti-inflammatory response. Studies demonstrate that EV-miRNA-mediated cell crosstalk between hepatocytes and macrophages after hypoxia treatment [51]. However, major eminent issues about the molecular mechanism in charge of EV-miRNAs modulate the duration and the magnitude of the immune response under pathological hypoxia are still partially unsolved.

## Relationship between EV-miRNA and inflammation under hypoxia

### MiRNA

MiRNAs belong to short non-coding RNA, which is a key element in cellular transcriptional regulation [52]. MiRNAs are transcribed into a primary miRNA transcript (pri-miRNA) and cut into a precursor miRNA (pre-miRNA), which can enter the cytoplasm to form mature miRNA, and eventually be directed to its targeted mRNA [53, 54]. In general, miRNA can bind to the target gene at mRNA at its 3′-untranslated regions (UTR) through incomplete pairing, which reduces the stability of mRNA or the translation of the target gene [55]. Importantly, they are involved in the regulation of almost all cellular processes, including cell differentiation, proliferation, apoptosis, especially signal transduction. Moreover, miRNAs regulate transcription in multiple ways, including but not limited to the specific interaction of miRNA-mRNA, targeting DNA sequence, regulation of miRNA localization and the synergistic effect of other miRNAs [56].

### Selective miRNAs loading in EVs under hypoxia

Compared with whole-cell miRNAs or free miRNAs, EV-miRNAs are considered to be more sensitive and specific due to they are selectively secreted into extracellular space. The abundant miRNA cargoes could lead to the greatest diversity of the downstream signaling. The sorting process of EV-miRNAs is not random, but highly selective, which can be regulated by specific endogenous target sequences. The short sequence motifs overexpression in miRNAs (EXO-motifs) in exosomes can regulate the sorting of miRNAs by promoting the combing of RNA-binding proteins (such as hnRNPA2B1, SYNCRIP) [57]. The heterogeneous nuclear ribonucleoproteins (hnRNPs) mediate miRNA packing into exosomes by recognizing GGAG motif [58, 59], and SYNCRIP can recognize the specific sequence (GCUG) at the 3′end of miRNA [60]. In addition, the miRNA induced silencing complex and membrane proteins are all involved in miRNA sorting process. The membrane proteins-dependent pathway is also one of the sorting mechanisms [61]. MiRNA sorting is a complicated phenomenon, and there is no definite and unified sorting mechanism yet, and further investigation is needed.

Another key question is how the miRNA sorting to EVs performs its biological function to the recipient cells. When EVs bind and load active mRNA and miRNA in the recipient cells, it is effectively regulated by De Novo-translation and post-translation regulation of target mRNA gene expression. EV-miRNAs in hepatocellular carcinoma cells promote tumor invasion and spread by inducing the expression of transforming growth factor-β(TGF-β) and TGF-β activated kinase-1 [62]. However, bone marrow-derived MSC can secrete miR-125-enriched exosomes, which can improve cardiac function by improving autophagy flux [63]. Together, the influence of EV-miRNAs on target cells depends on the selective loading of specific miRNAs and external stimulating factors, as well as the regulation of immune cells after binding to target gene segments of recipient cells.

### EV-miRNAs mediated inflammatory regulation

**Fig. 4:**
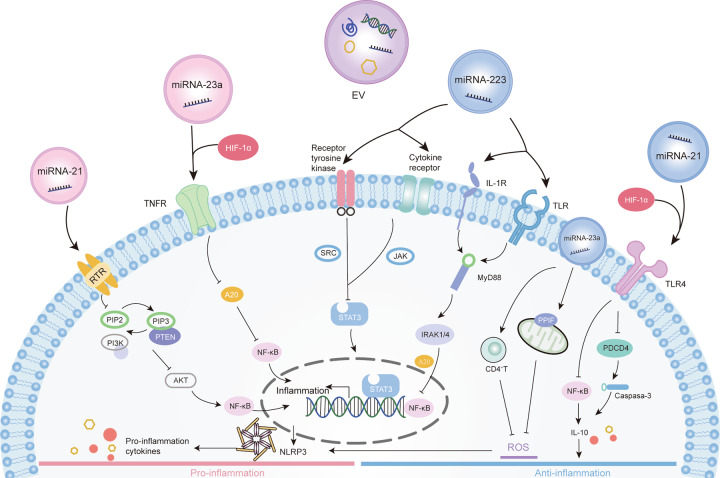
Mechanisms involved in the modulation of inflammation by miRNA derived EVs. In response to hypoxia, cells release extracellular vesicles (EVs) containing miRNAs, which regulate inflammatory signals. These signals can be detected by specific recognition receptors, thus altering the state of inflammation by affecting the associated signaling pathways and their downstream immune cell activation. MiR-223 can inhibit classical pro-inflammatory pathways through NF-κB and STAT3 pathway and enhances anti-inflammatory responses. It can suppress inflammation via inhibition of NLRP3 inflammasome activation. Under hypoxia conditions, the expression of miR-223 was downregulated and the anti-inflammatory effect was weakened. miR-23a-enriched exosomes mediated via HIF-1α that activate proinflammatory macrophages by inhibiting A20 target to NF-κB. It also can suppress reactive oxidative stress (ROS) and curbs necrosis by targeting peptidylprolyl isomerase F (PPIF). MiR-23a is essential for the proliferation of effector CD4^+^T cells and prevent tissue necrosis caused by ROS. MiR-21 plays an anti-inflammatory effect by inhibiting NF-κB and PDCD4/caspase-3 pathway. Conversely, miR-21 also participates in the positive feedback loop of inflammation through the inflammatory signals of NF-κB and STAT3 factor in tumor cells.

Plenty of studies have confirmed that some miRNAs are closely related to hypoxia and inflammation, and the most prominent are miR-233, miR-23a, and miR-21 (Fig. 4). A summary of EV-miRNAs and related macrophage changes is described in Table 1.

**Table 1:** Potential regulation and effects of EV-miRNAs on macrophage.

	Cell of origin	Species	Transferring materials	Biological effects	Target gene/single pathway	References
EVs	Bone marrow-derived MSCs	Human	miR-21-5p, miR-210-3p, miR-23a-3p	Promote M2-like polarization increased tumor growth, cancer cell proliferation, intra-tumoral angiogenesis	Downregulated PTEN, PDCD4 and RECK gene expression	[96]
Exosomes	Glioma	Human	miR-1246	Promote M2 macrophage polarization	Targeting TERF2IP via the STAT3 and NF-κB pathways	[124]
Exosomes	Adipocyte	Mice	miR-34a	Inhibit M2 macrophage polarization	Inhibiting the expression of Klf4	[5]
Exosomes	Tubular epithelial cells	Mice	miR-23a	Activates macrophages to promote tubulointerstitial inflammation	Suppression of A20	[27]
Exosomes	Neuron	Mice	miR-21-5p	Promote M1 microglia polarization	/	[87]
Exosomes	Tumor cell	Mouse Raw Human	let-7a miRNA	Promote M2 macrophage polarization	Suppression of the insulin-Akt-mTOR pathways	[126]
Microvesicles	Bone marrow-derived macrophages	Mice	miR-223/142	Suppresses macrophage activation	Inhibition of Nlrp3 inflammasome activation.	[68]

#### MiR-223

MiR-223 is a highly conserved anti-inflammatory miRNA during evolution, which is a post-transcriptional regulator of many genes necessary for inflammation, cell proliferation, and invasion. A list of genes, mainly including cancer and inflammation, that are validated as miR-223 targets [64, 65]. MiR-223 is a macrophage polarization regulator that inhibits classical pro-inflammatory pathways and enhances anti-inflammatory responses [66]. The Pknox1 is a real miR-223 target gene and a necessary regulator of macrophage polarization. MiR-223 can inhibit the activation of M1 macrophages and promote the expression of M2 markers by targeting Pknox1, which can prevent the inflammation of adipose tissue. The upregulation of miR-223 can reduce the production of IFN-γ, IL-6, and increase IL-10 [67, 68]. In the process of human monocyte-macrophage differentiation, the expression of miR-223, miR-15a, and miR-16 is significantly decreased, the inhibitory effect of NF-κB kinase α (IKKα) inhibitor was alleviated, and the signal transduction of NF-κB was enhanced, which led to the heighten of inflammation [69]. Furthermore, MVs can inhibit the activation of NLRP3(NLR family, pyrin domain containing3) inflammasome activation by transmitting miR-223/142 in stimulated cells, thus suppressing M1 macrophage activation [70]. Interestingly, due to the surface marker protein of MV-miRNA is same as that of parent cells, MV-miR-223 indicates the specific organ/location in which macrophages are activated, compared with non-MV-containing miR-223. Therefore, it also provides a novel method to predict the location and state of inflammation from EV-miRNAs. In general, miR-223 is one of the abundant miRNAs in EVs, the feature of its multiple functions is related to inhibition of inflammation-related genes, which can be used as a systemic homeostasis factor [53].

#### MiR-23a

Previous studies have shown that hypoxia can significantly induce the expression of miR-23a in different cells, especially relatively increased in primary macrophages [71]. Li et al. identified that miR-23a-enriched exosomes mediated via HIF-1α that activate macrophages by inhibiting the transmission of A20 to receptor macrophages in the process of inflammation [27]. A20, a ubiquitin editor, regulates the NF-κB pathway by certain ubiquitin from specific signal intermediaries, which can be inhibited to promote macrophage NF-κB activation. Conversely, the evidence demonstrates that miR-23a shows anti-inflammatory characteristics by inhibiting the increase of inflammatory bodies of NLRP3 [72]. As we all know, hypoxia can induce the production of ROS, and uncontrolled ROS can cause excessive inflammation and tissue damage [73]. MiR-23a can restrict ROS flux and maintain mitochondrial integrity via targeting the Peptidylprolyl Isomerase F(PPIF), the gatekeeper of the mitochondria permeability transition pore, and finally suppresses ROS and curbs necrosis. It is also essential for the proliferation of effector CD4^+^T cells and prevents tissue necrosis caused by ROS by providing early protection [74]. Previous evidence has shown that endoplasmic reticulum (ER) stress promotes hepatocellular carcinoma cells to release more miR-23a-p-enriched exosomes, which can damage T-cell function and lead to immune escape by up-regulating the expression of macrophage programmed death ligand1 through PTEN-AKT pathway [75]. Therefore, miRNA-23a-enriched exosomes were diverted to macrophages to regulate inflammation and tumor progression [76, 77].

#### MiRNA-21

MiR-21 is one of the most up-regulated miRNAs under hypoxic conditions, which is recognized by most people. It is a kind of anti-inflammatory miRNA, but it may have a pro-inflammatory effect in the process of cell transformation induced by oncogenes. The expression of miR-21 in macrophages has a negative regulatory effect on M1 macrophages [78], which is mainly through targeting programmed cell death 4(PDCD4) to increase the production of IL-10 [79]. Moreover, miR-21 plays a part in anti-inflammatory and anti-apoptosis by inhibiting NF-κB-TNF-α-TLR and PDCD4/caspase-3 pathway, respectively [80, 81]. Chen et al. confirmed that miR-21-5p protects monocytes in chronic intermittent hypoxia re-oxygenation by inhibiting the expression of inflammation-related genes [82]. Besides, the secretion of inflammatory cytokines (such as IL-6, IL-1β, TNF-α) and chemokine receptor7 also can be inhibited by miR-21 under hypoxia. Although HIF-1α is commonly known for its pro-inflammatory role, it can increase the expression of miR-21, which reduces inflammation and apoptosis by downregulating PDCD4 and Fas-L during ischemic postconditioning [83–86]. HIF-1α-induced upregulation of miR-21 expression may be a protective mechanism to adapt to hypoxia. However, the miRNA short-term upregulation is different from the overexpression of transfection and ultimately fails to counter the injury and inflammation caused by persistent hypoxia. These reactions are the process of cell adaptation contend with external stimulation.

However, the exosomes containing miR-21-5p can be phagocytized by microglia and induce M1 microglia polarization, which aggravates the release of neuroinflammatory cytokines and promotes cell apoptosis [87]. Meanwhile, it is also part of the positive feedback loop that links inflammation to cancer. When miR-21 is activated through the IL-6/STAT3 pathway, which can inhibit PTEN and increase the activity of NF-κB [88]. This inflammatory signal can initiate an epigenetic switch from nontransformed to cancer cells [89]. Studies have found that exosomes derived from PC12 cells are rich in miR-21 and can be delivered to normal cells, which can down-regulate the expression of TGFβRII and TPM1, thereby regulating cell transformation and tumor cell growth [90]. In addition, compared with non-activated macrophages, M2 macrophage-derived exosomes express higher levels of miR-21, which can confer drug resistance on these cells by metastasizing to gastric cancer cells [91]. The anti-inflammatory/pro-inflammatory effects of miR-21 may be caused by the characteristics of parental cells and the activation of signaling pathways in different receptor cells.

#### EV-miRNAs regulate the polarization of macrophages

The special role of miRNA carried by EV under hypoxia induces the polarization of macrophages by affecting key cytokines and the related molecular mechanisms are summarized in Table 2. Li et al. have reported that LPS-stimulated tumor cells can secrete MVs packaging miR-let-7b, which uptake into tumor-associated macrophage(TAMs) and inhibit M1 macrophage polarization to attenuates tumor inflammation by targeting IL-6 [92]. As a major pro-inflammatory mediator in cerebral ischemia, miR-3473b can be downregulated by IFN-γ, which promotes M1 macrophage polarization and targets PTEN to suppress the production of IL-10 [93, 94]. Moreover, the upregulation of miR-146a inhibit M1 macrophage activation through TNF-α-related receptors [95]. Hypoxic-driven EV-miR-21 can stimulate increased gene expression associated with M2 macrophages by inhibiting PGE2 [96]. Furthermore, the miR-21 also increased M2 macrophages by participating in downstream action of colony-stimulating factor 1 receptor (CSF-1R) [97]. MiR-23a/27a/24-5p cluster was found to be lowered in TAMs through different but related pathways [98]. They inhibit IL-4-stimulated M2 polarization via suppressing the JAK1/STAT6 pathway. The downregulation of hypoxic-driven EV-miR-223 active STAT3 to increase IL-6 and IL-1β production, thus inducing polarization of M2 macrophages [99]. The above evidence suggests that the function of cytokines is an important link in the mediators of EV-miRNA regulating macrophage polarization. In general, the identification of miRNA targets in different diseases and cell types will give us a better understanding of the mechanism of their influence on macrophage polarization and display valuable in applying miRNA therapeutics with high confidence.

**Table 2:** Key miRNAs involved in macrophage differentiation and modulation of pro/anti-inflammatory polarization.

miRNA	Key regulatory factors	Biological effects	Polarization of macrophages	References
miR-3	IL-6	Enhance the secretion of IL-6 by inhibiting the JAK/STAT signaling pathway	Promote M1 macrophage polarization	[121]
miR-3473b	IFN-γ	Downregulated by IFN-γ and targets PTEN to suppress the production of Akt signaling and IL-10	Promote M1 macrophage polarization	[94]
miR-146a	TNF-α	Indirect regulation of multiple components of TNF-α signaling pathways (such as MyD88,TRAF6)	Inhibit M1 macrophage polarization	[95]
miR-let-7b	IL-6	Inhibits IL-6 through the TLR signaling by a MV-based model	Inhibit M1 macrophage polarization	[92]
miR-23a/27a/24-5p	IL-4	Inhibit IL-4- via suppressing the JAK1/STAT6 pathway or targets IRF4 and PPAR-γ	Inhibit M2 macrophage polarization	[98]
miR-223	IL-10	Reduce the production of IFN-γ, IL-6 and increase IL-10 by targeting Pknox1	Promote M2 macrophage polarization	[68]
miR-21	PGE2	Inhibiting PGE2 via target STAT3	Promote M2 macrophage polarization	[96]

### Signaling pathway of EVs involved in inflammation

As an important mediator of hypoxia and inflammatory crosstalk, the changes of EV-miRNAs are regulated by various signal pathways. Here we mainly describe the most prominent signal pathways, such as NF-κB, TLR, STAT3, which associate EVs with inflammatory events (Fig. 4).

#### NF-κB signaling

Members of the NF-κB transcription factor family participate in cell differentiation and proliferation, inflammatory and immune response [2]. Activation of NF-κB depends on the degradation of the inhibitor of NF-κB (IκB) proteins [100]. There are two types of NF-κB signaling pathways: canonical and noncanonical pathways. The canonical pathway can be activated rapidly and briefly, while the noncanonical pathway is activated relatively slowly due to the depend on de novo synthesis of NF­κB­inducing kinase [101]. The target genes of canonical NF-κB signaling mainly include pro-inflammatory factors, chemokines, anti-apoptotic proteins, and inducible nitric oxide synthase (iNOS). Activation of NF-κB was further regulated by EV-miRNAs, such as miR-146, miR-21, either positively or negatively. Exosomes derived from dendritic cells participate in endothelial inflammation through the NF-κB pathway mediated by TNF-α [102]. Excessive activation of NF-κB is closely related to the state of tissue inflammation. MiRNA-138 has been observed to induce inflammation and apoptosis through exosomes mediated via the VEGF/NF‑κB pathway [103]. Studies have reported that melatonin reduces the inflammation of adipocytes by transporting exosomal α-ketoglutarate(αKG) to macrophages, which promote DNA demethylation and M2 macrophages polarization through weakening STAT3/NF-κB pathway [104]. Meanwhile, the hydroxylase that regulates HIF also gives hypoxia sensitivity to the NF-κB pathway through oxygen-dependent hydroxylation of key components [2, 105, 106]. Activation of NF-κB and nuclear translocation are necessary for hypoxia tolerance [107]. The special role of HIF‐1α up-regulating the expression of NF‐κB to promote the release of inflammatory cytokines in macrophages has been observed [108]. However, the majority of previous research has focused on the mechanism of HIF-NF-κB pathway are focused on HIF-1α rather than HIF-1β and HIF-2α in inflammatory conditions, both of which are related to activity of NF-κB.

#### TLR4 signaling

Toll-like receptors (TLRs) are type I transmembrane proteins and a group of pattern recognition receptors of immune system that are involved in inflammation via endogenous ligand detection [109]. As a member of TLR family, TLR4 contains a Toll/IL-1R homology (TIR) domain responsible for signal transmission intracellular and a module structure of the extracellular that connected with the TIR. After molecular recognition of circulating LPS, receptor dimerization is carried out on the cell membrane, which will induce the protein-protein cascade reaction [110]. HIF-1α binds to the promoter region of TLR4 under hypoxia, which promotes the accumulation of TLR4 mRNA and protein in macrophages. The upregulation of TLR4 expression enhances the response of macrophages to LPS, leading to more cyclooxygenase-2 and IL-6 expression, which enhances sensitivity and the ability to respond to inflammatory signals [111]. However, Ishida et al. identified that hypoxia can reduce the expression of TLR4 in cultured endothelial cells [112], which may be due to the decrease of ROS-mediated activate protein-1 binding activity [113]. Interestingly, a close interaction between TLRs and miRNAs had been found. EV-miRNAs affect the expression level of TLR and the intensity of the inflammatory reaction [114]. MSC-exosomes-miR-182 polarize macrophages from M1 to M2 phenotype in vivo and in vitro, with TLR4 as a downstream target [115]. EV-miRNAs have been observed to reduce LPS-induced inflammation and apoptosis by inhibiting TLR4/NF-κB and p53 pathways in intestinal epithelial cells [116]. Therefore, it may be a feasible strategy to regulate the inflammatory process by manipulating the expression of TLR4 to treat inflammation-related diseases.

#### STAT3 signaling

Signal transducers and activators of transcription (STAT) are a kind of cellular signal transcription factor, which participates in the regulation of cell differentiation, proliferation, angiogenesis. STATs were considered as potential cytoplasmic transcription factors that can be phosphorylated by the Janus Kinase (JAK) tyrosine family after stimulation by a variety of cytokines. Phosphorylation induces STAT dimerization and translocation to the nucleus, where it binds to specific promoters to regulate transcription [117]. Since NF-κB acts as a core transcription factor in multiple immune responses, it is not surprising that it is highly correlated with STAT3 signaling. STAT3 has been well-explored and plays a dual role in regulation of tumor inflammation [118]. It is promoting oncogenic inflammation through the NF-κB-IL-6-JAK pathway or suppressing immune responses by antagonizing STAT1 and NF-κB-mediated T helper cell1, respectively. Hypoxia is often present in macrophages at the sites of inflammation and infection, accompanied by high expression of TNF, which inhibits the expression of the IL-10-STAT3 autocrine loop and later phases inflammation-induced degradation genes (e.g., NF-κB and TLR inhibitors). Therefore, the inhibition of IL-10-STAT3 is one of the molecular mechanisms by which hypoxia promotes inflammation through macrophages [119]. Studies have confirmed that exosomes derived from breast epithelial cells tend to regulate the activation of M1 macrophages by enhancing the expression of miR-221, which is mainly accomplished by inhibiting the SOCS1 cascade and regulating STAT1 and STAT3 [120]. In addition, exosomes containing miR-3 promote the polarization of M1 macrophages, and enhance the secretion of IL-6 by inhibiting the JAK/STAT signaling pathway [121]. In general, STAT3 is a potential therapeutic target for restrict inflammation for disease therapy.

## EVs-miRNA, macrophage, tumor

As the most abundant immune cells in tumor microenvironment, high macrophage density is associated with poor prognosis in a variety of tumor types, so it is necessary to work on the plasticity and polarization of macrophages [122]. However, it is worth mentioning that while M2 macrophages promote tumor growth and metastasis, M1 macrophages also play an important role in tumorigenesis and cancer. In chronic inflammatory environments, M1 macrophages promote the induction of oncogenic processes by increasing the secretion of pro-inflammatory mediators, but they counteract the growth of established tumors by stimulating anti-tumor immune responses and direct tumoricidal activity [123]. Most of all, EV-miRNAs participate in regulating macrophage polarization, as integral components of feedback loop regulatory mechanisms, through binding interactions with several key transcription factors. Qian et al. [124] identified that hypoxia glioma-derived exosomes significantly induce the polarization of M2 macrophages compared with normoxic glioma-derived exosomes, and further confirmed that this process is accomplished by activating STAT3 pathway via delivering miR-1246. Besides, MSC-EV transmission miR-21-5p to receptor cells after hypoxia, which promotes the development of lung cancer by promoting polarization of M2 phenotype and reducing apoptosis macrophages [125]. Park et al. identified that exosome-derived tumor cells are enriched in immunomodulatory proteins and chemokines, which can affect the recruitment of macrophages and promote M2-like polarization, thus further evading host immunity through oxidative phosphorylation [126]. At present, the polarization of M2-like macrophages and the changes of immune metabolism are considered to be an important part of tumor progression but it is still a long way to explore the exact mechanism, and it is also an attractive target for new immunotherapy.

## Conclusion and future directions

Hypoxia and inflammation are intertwined at the molecular, cellular, and clinical levels. EV-miRNAs plays a key role in cellular communication in hypoxia-induced inflammation, mainly by controlling the polarization of macrophages between M1 to M2 phenotypic. Some miRNAs lead to the development of innate responses and the secretion of pro-inflammatory cytokines, while others negatively control the inflammatory response to prevent excessive inflammation. Adopting an appropriate strategy for manipulating the expression of miRNA while keeping our eyes open to the potential interactions between various miRNAs, may have therapeutic potential in the treatment of hypoxic inflammation-related diseases. Future studies are greatly needed to test whether these EV-miRNAs can be used in clinical. In addition, the mechanism of change of formation, cargo sorting, and secreting of EVs under hypoxia and how EV-miRNAs affecting inflammation need more attention.

A new application is the use of EVs as carriers to deliver specific compounds to regulate cell function in vivo. Owing to EVs characterized by good stability, small size, slow cycle clearance, and strong loading capacity, especially its biocompatibility, which is unlikely to trigger innate and acquired immune responses, becoming an attractive tool for therapeutic molecular delivery. Also, specific shipments (e.g., interfering RNA, suicide mRNA/ proteins, miRNA, and drugs) are loaded based on in vitro manipulation of the EVs and then delivered to the target cell as a drug or for bioengineering purposes [11, 127]. Regulating the specificity of EVs targeting receptor cells will be the key to their use as high-precision carriers. These characteristics show the potential of EVs in the coming therapeutic application.

## Data Availability

All data generated or analyzed during this study are included in this published article.
